# Faecal Cyathostomin Egg Count distribution and efficacy of anthelmintics against cyathostomins in Italy: a matter of geography?

**DOI:** 10.1186/1756-3305-2-S2-S4

**Published:** 2009-09-25

**Authors:** Piermarino Milillo, Albert Boeckh, Rami Cobb, Domenico Otranto, Riccardo P Lia, Stefania Perrucci, Antonio Frangipane di Regalbono, Paola Beraldo, Georg von Samson-Himmelstjerna, Janina Demeler, Roberto Bartolini, Donato Traversa

**Affiliations:** 1 Faculty of Veterinary Medicine, University of Teramo, Italy; 2 Fort Dodge Animal Health, USA; 3 Faculty of Veterinary Medicine, University of Bari, Italy; 4 Faculty of Veterinary Medicine, University of Pisa, Italy; 5 Faculty of Veterinary Medicine, University of Padua, Italy; 6 Faculty of Veterinary Medicine, University of Udine, Italy; 7 University of Veterinary Medicine, Hannover, Germany

## Abstract

**Background:**

In the framework of a trial carried out in 2008 in Europe to evaluate the efficacy of major parasiticides against horse cyathostomins, pre- and/or post-treatment Faecal Egg Counts (FEC) were evaluated in a total of 84 yards and 2105 horses from nine different regions from the South, the Center, the North-Center and North-East of Italy. Specifically, on the basis of FECs of the horses present in each property, 60 out of the 84 yards were enrolled for a Faecal Egg Count Reduction Test (FECRT) using fenbendazole, pyrantel, ivermectin and moxidectin.

**Results:**

Of the 1646 horses bred in the 60 recruited yards, 416 animals had a FEC between 50 and 150 Eggs Per Gram (EPG) of faeces and 694 a FEC >150 EPG (i.e. with total of 1110 positive animals). Of the 1110 positive animals, those with the highest FECs (i.e. 988) were included in the FECRT. The FECRT for four anthelmintic compounds showed remarkable differences in terms of prevalence of reduced and equivocal efficacy against cyathostomins in the different areas of Italy. Administration of fenbendazole and pyrantel resulted in resistance present or suspected in about half of the yards examined while resistance to ivermectin was found in one yard from central Italy and suspected resistance was detected in three more yards, one in each the North, the Center and the South. Treatment with moxidectin was 100% effective in all yards examined.

**Conclusion:**

Cyathostomin populations in the South and the Center of Italy were more susceptible to fenbendazole and pyrantel than the populations present in the Center-North and North-Eastern areas of Italy. Fenbendazole and/or pyrantel were ineffective in almost all properties from the North of Italy. The reasons for such a difference among the Italian regions in terms of FECs and efficacy of antiparasitic drugs are discussed, together with the role that veterinarians, and horse owners and managers should have for effective worm control programs in this country.

## Background

Accurate information on the spread and biology of horse cyathostomins in Italy is scanty and fragmentary though their importance is recognized in equine current practice and populations resistant to the common anthelmintic compounds have been reported in many parts of the world [1]. Infection by cyathostomins has been reported in different Italian regions, e.g. in the North in Lumbardy [2] and Friuli Venzia Giulia [3] regions, and in the Center in Marche [4] and Umbria [5] regions, with prevalence rates up to 100%. Recent studies have also demonstrated the presence of populations resistant to one or more anthelmintics in different regions of the Country [6,7]. A broad study carried out in 2008 has enhanced our knowledge on the presence and diffusion of drug-resistant cyathostomin populations in a large area of the Italian territory. From a total of 84 yards evaluated, only 60 had sufficient number of horses positive by FEC for enrollment in the survey. In these 60 yards, 1646 horses screened by the FEC for the presence of cyathostomins and a total of 988 animals were subjected to a Faecal Egg Count Reduction Test (FECRT) to evaluate the efficacy of the major anthelmintic compounds used in current practice against cyathostomins in our country [7,8]. These yards were located in geographically and socially distinct areas in Southern, Central, Central-Northern and North-Eastern Italy [7,8] and the results found showed differences in both FECs prior to treatment and the efficacy of antiparasitic compounds in the different regions of Italy included in the trial.

Given that information on the epidemiological differences and on the efficacy of antiparasitic drugs against cyathostomins in regions within a country is key to the implementation of effective control measures and for preventing the spread of resistant populations, the aim of the present article is to report and discuss separately such differences in FECs and efficacy of anthelmintic compounds against horse cyathostomins in four different geographical settings of Italy.

## Methods

### Yards and animals

The presence of cyathostomin infection was evaluated in a total of 84 yards and 2105 horses in Italy by faecal egg counts (FEC) on all animals present on each of the properties [8]. A range of different types of farms participated in the survey, including pleasure, sport and training facilities. No animals bred for slaughtering purposes were included in the trial.

On the basis of FECs of the horses present in each property, 60 out of the 84 yards were enrolled for a Faecal Egg Count Reduction Test (FECRT). In particular, a minimum of 12 horses with ≥ 50 eggs (cut-off value) of cyathostomins per gram of faeces (EPG) was the criteria used to enrol yards in the FECRT from 9 different Italian regions (Table 1). Of the 1646 horses on the whole bred in the 60 yards, animals with the highest FECs (i.e. 988) were subjected to the FECRT.

**Table 1:** Number of horse yards enrolled (Ye) in the Faecal Egg Count Reduction Test in nine regions of Italy. Ht: horse treated in enrolled yards. Hd: horse not treated (i.e. discarded) in enrolled yards.

**Region**	**Ye**	**Ht**	**Hd**
*Southern Italy*			
Calabria	6	108	36
Puglia	8	136	61
Basilicata	6	88	35
*Central Italy*			
Lazio	9	164	60
Abruzzo	9	148	127
Umbria	2	28	36
*Central-Northern Italy*			
Toscana	10	160	191
*Northern-Eastern Italy*			
Friuli-Venezia Giulia	5	64	77
Veneto	5	92	35
*Total*	60	988	658

### Faecal Egg Count Reduction Test

These 988 horses were subjected to a Faecal Egg Count Reduction Test (FECRT) evaluating the efficacy of fenbendazole (FBZ), pyrantel (PYR), ivermectin (IVM) or moxidectin (MOX) as previously described [7]. Briefly, on Day 0, horses enrolled in each treatment group were treated with either FBZ, PYR, IVM or MOX. Individual faecal samples were collected for each animal at dosing on Day 0 and on Day 14 to determine the individual pre-treatment and post-treatment EPG values. Faeces were subjected to a quantitative coproscopic analysis and arithmetic means of the pre- and post-treatment faecal egg counts were used to calculate the group FECR according to the formula FECR = 100 * (1 - FEC post-treatment/FEC pre-treatment). The lower and upper 95% confidence limits using a re-sampling number of 2000 were calculated. The obtained FECRs were categorized for all tested compounds as follows: (I) *resistance present *if FECR <90% and the lower 95% confidence limit (LCL) <90%, (II) *resistance suspected *if FECR ≥ 90% and/or LCL <90% and (III) *no resistance *if FECR ≥ 90% and LCL >90%.

For the comparative investigation of cyathostomin egg counts in different geographical regions all pre-treatment individual FECs were conventionally divided in three FEC categories:

• negative FEC (*no shedders*)

• FEC from 50 to 150 EPG (*low shedders*)

• FEC >150 EPG (*high shedders*)

Post-treatment larval cultures from each yard were performed from pooled faecal samples collected from each treatment group. Faeces were mixed with oak sawdust and water. Coprocultures were incubated for 10 days at 27°C and after incubation third-stage larvae (L3) were harvested using the baermann technique, examined using a light microscope and identified using morphological keys [9].

## Results

**Figure 1:**
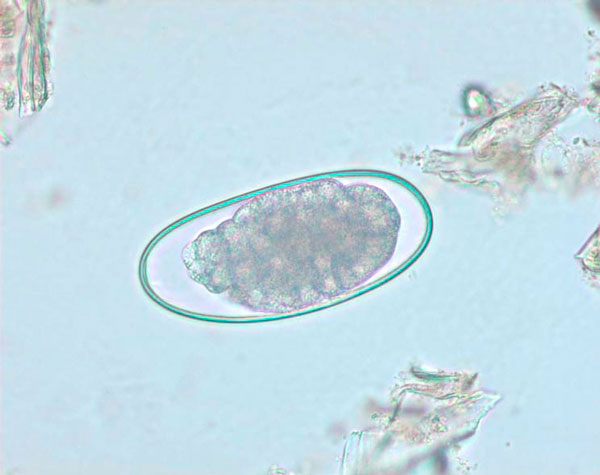
**Egg of cyathostomins**.

No yards negative for cyathostomins were found. Sixty (60) out of 84 (71.4%) properties had a minimum of 12 horses with a ≥ 50 EPG cyathostomin FEC (Figure 1), which were recruited for the FECRT.

**Figure 2:**
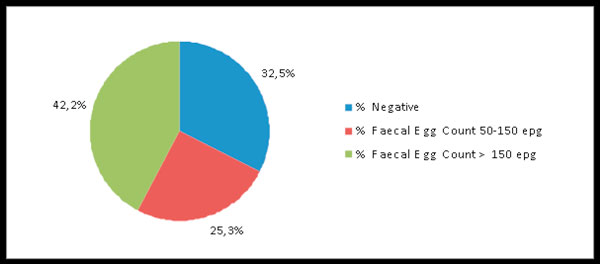
**Percentage of horses negative to cyathostomin eggs (*no shedders*), horses with a Faecal Egg Count from 50 to 150 cyathostomin Eggs Per Gram of faeces (*low shedders*) and horses with a Faecal Egg Count >150 cyathostomin Eggs Per Gram of faeces (*high shedders*) on a total of 1646 animals examined in nine regions of Italy**. The 1646 animals were overall kept in 60 yards enrolled for a Faecal Egg Count Reduction Test.

Of the 1646 horses examined copromicroscopically in the 60 yards recruited for the FECRT, 1110 (67.4%) were infected by small strongyles (536 resulted negative). Of the 1110 positive horses, 416 (25.3%) had FEC between 50 and 150 EPG and 694 (42.2%) FEC >150 (Figure 2). Table 2 reports the number of horses negative for cyathostomins, with a FEC from 50 to 150 cyathostomin EPG and with a FEC >150 EPG on the total of 1646 animals kept in the 60 yards enrolled in the FECRT.

**Table 2:** Number of horses negative for cyathostomins (N), with a Faecal Egg Count from 50 to 150 cyathostomin Eggs Per Gram of faeces and with a Faecal Egg Count >150 cyathostomin Eggs Per Gram of faeces on a total of 1646 animals examined in Southern, Central and Northern Italy.

	**N**	**50-150**	**150**	**Total**
South	95	180	189	464
Center	188	131	244	563
North	253	105	261	619
Total	536	416	694	1646

In the North of Italy the highest percentage of *high shedders *in a single property was detected in yard number 4 from Friuli Venezia Giulia (82%), whereas in yard number 2 of the same region the highest proportion of *low shedders *(51.4%) in this area of the country was found. The lowest presence of *high shedders *and the highest percentage of negative animals were found in Toscana with 15% and 70.4%, respectively, of the horses present in yard number 1. The lowest rate of *no shedding *animals was found in yard number 4 from Friuli Venezia Giulia (5.7%).

In central Italy the percentage of *high shedders *ranged from 14.7% in yard number 6 from Abruzzo to 90% in yard number 6 from Lazio. In the same yards the maximum and minimum rate of low shedders have been detected, i.e. 52.9% and 10% respectively. No negative animals were found in yards nos. 1-6 from Lazio and in yards number 7 and number 9 from Abruzzo. Yard number 7 in Lazio region showed the highest percentage of negative horses (66%).

With regard to Southern Italy, all horses (100%) tested in yards number 1 and number 8 from Puglia and in yards number 1, number 5 and number 6 from Calabria were positive. Yard number 1 from Puglia had the highest percentage of *high shedders *(85%), while the lowest (11.1%) was found in yard number 2 from Calabria. The highest rate (68.8%) of *low shedders *was found in yard number 8 of the former region, while the lowest was registered in yard number 3 from Basilicata.

As far as the results of the anthelmintic efficacy evaluation, all four compounds were tested in 50 yards, where 12-20 sufficient cyathostomin infected horses were available. In 10 properties only PYR, IVM and MOX were evaluated in three treatment groups of four animals each due to the availability of FEC positive horses. Of the positive horses, 988 were overall included in the FECRT, i.e. animals with the highest pre-treatment FECs (Table 1).

Administration of FBZ resulted in *no resistance *in about half of the examined yards, i.e. 23 (46%), while *resistance present *was detected in 19 (38%) yards, and *resistance suspected *in 8 (16%) yards.

*Resistance present *and *resistance suspected *for PYR were found in 18 (30%) and 17 (28.3%) yards respectively, while in the remaining 25 (41.7%) this molecule was active against small strongyles.

Cyathostomin populations resistant to IVM were found in 1 yard (1.7%), specifically in yard number 1 of Abruzzo. The administration of this compound gave results of *resistance suspected *in 3 (5%) more yards, i.e. yard number 5 from Calabria, yard. 3 from Lazio and yard number 2 from Veneto, and *no resistance *in the remaining 56 (93.3%).

Treatment with MOX was 100% effective in all yards examined.

Occurrence of multiple resistance has been found on 10 out of 50 yards (20%), i.e. nine for FBZ + PYR and one for FBZ + IVM.

Evaluation of results for individual horses, showed 0-100% range of efficacy for FBZ, PYR and IVM, while MOX displayed 100% efficacy in all treated yards.

The FECR for the four compounds showed remarkable differences in terms of prevalence of reduced and equivocal efficacy in the different geographic areas of Italy. In fact, the cyathostomin populations in the South and the Center were found to be more susceptible to FBZ and PYR than the populations present in the Center-North and North-Eastern areas of the peninsula (Table 3). In fact, in the Center of Italy, resistance to FBZ was detected in almost all yards from Lazio (7 out of 9) while in Abruzzo and Umbria the efficacy of the same compound was reduced or equivocal in only three out of ten properties. FBZ and PYR were found to be ineffective in almost all properties from the northernmost regions of Italy and, in particular, FBZ was not effective in any yard from the area and PYR effective in only one. In all but one yard from the same area multiple resistance to FBZ and PYR was found (Table 3).

**Table 3:** Percentage of horse yards (Y) with cyathostomins showing resistance (R) or suspected resistance (Rs) to fenbendazole (FBZ), pyrantel (PYR), ivermectin (IVM) and moxidectin (MOX).

	**% - R/Rs**			
	
**Y**	**FBZ**	**PYR**	**IVM**	**MOX**
South	12.5/25	15/30	0/5	0/0
Center	44.4/11.1	15/30	5/5	0/0
Center-North	30/20	60/20	0/0	0/0
North	100/0	60/30	0/10	0/0

## Discussion and conclusion

This survey demonstrated that resistant cyathostomin populations to FBZ and PYR are present in Italy, with different occurrence rates according to the four areas included. Worthy of note are also the reports of reduced efficacy for IVM (i.e. one yard with <90% efficacy and three more properties with <100% efficacy) and the presence of parasitic populations with limited susceptibility to more than one anthelmintic compound. These results are the first evidence for reduced efficacy of IVM in controlling small strongyles in Italy [2-5]. Interestingly in 2007 cases of <100% efficacy of ivermectin were found in Italy [6], that now can be considered as earlier indications of developing resistance to IVM in Italian pupulations of small strongyles. Conversely, MOX was found to be 100% effective in treating the infection caused by cyathostomins in all regions tested [7,8].

With regard to the differences of drug efficacy in different areas, both FBZ and PYR showed variable ranges of activity in the regions included in the study.

The administration of FBZ in North-Eastern Italy was ineffective in all tested yards and levels of reduced/absent efficacy of PYR were detected in 9 out of 10 properties, indicating that cyathostomin populations resistant to benzimidazoles (BZs) and also tetrahydropirimidines

(THP) are consistently present in the investigated area. Moreover, a single-dose of macrocyclic lactones (MLs) was effective (FECR = 100%) in all examined yards, with the only exception of one property where the efficacy of IVM was 94%.

Both the prevalence of horses shedding cyathostomin eggs and the occurrence of resistance to commonly used products mean that currently administered worm control programmes are not providing optimal control of worm parasites, even in operations where horses are valued and cared for by their owners.

Factors to consider in understanding the basis of the high prevalence of resistance to FBZ and PYR detected in this survey in north-eastern Italy could include prior anthelmintic treatment practices and factors such as underdosing and over-use of products. The life cycle of small strongyles may be poorly understood by horse owners, many of whom implement worm control programmes without input from veterinarians. As a consequence there is an erroneous perception of owners to that more frequent treatments may be beneficial in protecting animals from strongyles than are actually needed based on the life cycle and epidemiology. Yards with detected or suspected resistance reported administering four or more anthelmintic treatments to their horses annually. Treatment of all horses at the same time is a common practice. This management approach of the owners may particularly be due to the fact that the vast majority of horses bred in the North of Italy are for pleasure and sport, thus with high economic value and a focus on performance. Worm infestation was recognised as having detrimental effects. Moreover, in the investigated farms the removal of faeces from paddocks and mixed grazing with other animals are not carried out by breeders, both of which have been recommended as techniques for reducing worm challenge and thus reducing the necessity for frequent anthelmintic treatments. Conversely, *off label *use of anthelmintic treatment and/or daily feeding of low dose of THPs were not reported by the owners so these practices can be excluded as contributing to the higher prevalence of anthelmintic resistance found in this region.

An interesting observation is that single or multiple resistance to FBZ and/or PYR was seen in yards (6/10) with a higher percentage (>50%) of animals shedding high levels of faecal egg counts (>150 epg), while resistance only to FBZ was observed in one out of two yards with lower proportion (<1/3) of low egg shedders (≤ 150 epg). These results confirm that blanket treatments without knowing the resistance status of worms is not a good strategy for control and highlight the importance to promote routine FECs and veterinary advice to identify the horses needing an anthelmintic treatment, in order to assure both horse health and the maintenance of susceptible *refugia*. Horses identified as low shedders will not require treatment as frequently as those in the high shedding category. These horses, if known as carriers of drug-susceptible nematodes, when left untreated at a time when others in the herd require anthelmintics, will provide a source of eggs from unexposed nematodes to provide a useful dilution of resistant cyathostomin genotypes on pasture, without contributing significantly to increasing pasture contamination and overall challenge. In this survey, owners did not report the use of screening FECs to obtain guidance for possible control programmes against cyathostomin infections. However, in yards where the greater part of the cyathostomin population is present in a high proportion of animals and resistance to FBZ and/or THPs is detected/suspected, it is essential to avoid use of FBZ and/or PYR. The choices of alternative treatments are limited, and strategies to preserve the efficacy of MLs must be implemented as a matter of priority. Little is known about the mechanisms of resistance of cyathostomins to the MLs in horses, although published investigations into such mechanisms in strongyle parasites of ruminants show there are marked differences in rates of selection, and both quantitative and qualitative genetic changes resulting from exposure to IVM vs. MOX. For instance, selection for resistance in *Haemonchus contortus *in sheep was shown to occur more quickly to IVM than MOX when the parasite was exposed to sub-therapeutic levels of either compound, and IVM resistant parasites also showed reduced susceptibility to MOX, although this compound remained fully effective at the recommended commercial dose rate. IVM has been shown to be a substrate for P-glycoproteins, a recognized pathway used by other drug resistant pathways, while MOX is significantly less susceptible to P-glycoprotein transport, by orders of magnitude. IVM-resistant strongyle parasites have been shown to over express P-glycoproteins. The complexity of ML resistance has been further highlighted by recent publications that have identified a possible relationship between macrocyclic lactone and benzimidazole anthelmintic selection [10-20].

The seriousness of concerns about anthelmintic resistance has led to some speculation that MOX-based anthelmintics should be held in reserve, and used only as a last resort to control parasites only after IVM resistance has occurred. In the absence of any information to indicate ML resistance in equine small strongyles is mechanistically different from the mechanisms identified in ruminant strongyles, such speculative advice is likely to lead to accelerated selection for resistance to both available ML compounds. The preferred option based on current state of scientific knowledge is to use MOX appropriately, taking into account the extended recommended re-treatment interval which allows effective worm control through fewer treatments than would be required with IVM. The importance of treatment frequency in the selection for resistant cyathostomins was re-enforced by the findings in regions of Center and South, as discussed below.

In central Italy it is interesting to note that while FBZ was ineffective in almost all yards from Lazio region, this was not the case in the other two regions. This apparent discrepancy may be accounted for by the fact that, similarly to the South of the Country (see below), several horse yards in Abruzzo and Umbria do not usually perform a control worm program and/or have animals for slaughtering purposes. Thus, the parasitic populations in this territory are not subjected to a high selection pressure by anthelmintic drugs. Conversely, almost all properties from Lazio region were infected by FBZ-resistant cyathostomins and this can be due to the fact that the owners and veterinarians of the region had widely adopted the program to treat all the animals present on the property at least 6 times per year without any FECs before, thus promoting the pressure for the drug-resistance [6]. This is mainly due to the erroneous perception that the number of treatments is related to the health and welfare of the horses, especially in sport, training and breeding facilities, that are diffused in Lazio region of central Italy. The low efficacy of the widely used FBZ is reflected also by the fact that in 6 out of 9 yards from Lazio regions no animals resulted negative for cyathostomin eggs. Repeated treatment with a compound that cyathostomins are resistant to does not benefit the horse but simply results in increasing pasture contamination, increased resistance levels and worm challenge.

With regard to Southern Italy, percentages of resistance to FBZ and PYR showed lower rates than found in the other regions examined in Italy. Such a difference may be explained by the fact that, usually, in the southermost regions of our country farmers and veterinarians administer an anthelmintic compound only once or twice per year. Moreover, horses are imported in the South of Italy from different countries of Eastern Europe (e.g. Bulgaria, Rumania, Poland and Montenegro) for slaughtering purposes and meat consumption. While some of these imported horses are immediately destined for the abattoirs, in most cases the majority of the remainder (especially foals and yearlings) are locally held on properties for about 4-8 months without any anthelmintic treatment. Therefore, such a picture could lead to the hypothesis that the cyathostomin populations present in Southern Italy are subjected to a low selection pressure due to the lower use of antiparasitic treatments and that the small strongyle populations present on pasture and in horse yards of the south of Italy are composed of parasitic individuals mostly susceptible to the different parasiticides.

In conclusion, the situation depicted by the analysis of the FECs and FECRT in four different geographical areas of Italy demonstrates that the distribution of cyathostomins resistant to anthelmintic compounds is different in the different regions. The results are representative of the general situation in the surveyed regions, as yards and properties that were enrolled in the study are representative of the general social and economic situation of the region where they are located.

The regional variations in the results are likely due to differences in terms of practice management by owners and veterinarians and in the use of anthelmintics in the different areas even though further studies are warranted to confirm these differences. Therefore, it is crucial that owners, managers and veterinarians from Italy should be aware of the biological and epidemiological features of the infection by small strongyles in their regions, in order to plan appropriate worm control programs in the animals bred in the managed yards.

## Competing interests

Fort Dodge Animal Health provided financial and logistic support to this study. The authors declare that there are no competing interests and that the conceptual design, the conduct, the interpretation or any other scientific aspect of the study have not been influenced by the FDAH support.

## Authors' contributions

PM: Participated in the faecal sampling, coordinated and conducted the copromicroscopical analysis and contributed in drafting the manuscript. AB: Initiated the study and contributed to the design of the study. RC: Initiated the study and contributed to the design of the study. DO: Participated in yards enrolment and faecal sampling. RPL: Participated in yards enrolment and faecal sampling. SP: Participated in yards enrollment and faecal sampling. AFdR: Participated in yards enrollment and faecal sampling. PB: Participated in yards enrollment and faecal sampling. GvSH and JD: Contributed to the design of the study, participated in the statistical analysis and in drafting the manuscript. RB: Participated in the copromicroscopical analysis. DT: Contributed to the design of the study, was responsible for the study and for the copromicroscopical analysis of all faecal samples, and drafted the manuscript
